# Ligand activation induces different conformational changes in CXCR3 receptor isoforms as evidenced by plasmon waveguide resonance (PWR)

**DOI:** 10.1038/s41598-017-11151-x

**Published:** 2017-09-06

**Authors:** K. Boyé, C. Billottet, N. Pujol, I. D. Alves, A. Bikfalvi

**Affiliations:** 1 INSERM, U1029 Pessac, France; 2 Université de Bordeaux, Pessac, France; 3 CBMN, UMR 5248 CNRS Pessac, France

## Abstract

The chemokine receptor CXCR3 plays important roles in angiogenesis, inflammation and cancer. Activation studies and biological functions of CXCR3 are complex due to the presence of spliced isoforms. CXCR3-A is known as a pro-tumor receptor whereas CXCR3-B exhibits anti-tumor properties. Here, we focused on the conformational change of CXCR3-A and CXCR3-B after agonist or antagonist binding using Plasmon Waveguide Resonance (PWR). Agonist stimulation induced an anisotropic response with very distinct conformational changes for the two isoforms. The CXCR3 agonist bound CXCR3-A with higher affinity than CXCR3-B. Using various concentrations of SCH546738, a CXCR3 specific inhibitor, we demonstrated that low SCH546738 concentrations (≤1 nM) efficiently inhibited CXCR3-A but not CXCR3-B’s conformational change and activation. This was confirmed by both, biophysical and biological methods. Taken together, our study demonstrates differences in the behavior of CXCR3-A and CXCR3-B upon ligand activation and antagonist inhibition which may be of relevance for further studies aimed at specifically inhibiting the CXCR3A isoform.

## Introduction

The chemokine receptor CXCR3 belongs to the G-protein-coupled receptor (GPCR) family. CXCR3 regulates several biological functions and plays important roles in angiogenesis, inflammation and cancer^1–6^. Three distinct CXCR3 spliced isoforms have been described including CXCR3-A, CXCR3-B and CXCR3-alt. Theses isoforms differ in their carboxy- or amino-terminal domains. In particular, CXCR3-B has an amino-terminal domain 52 amino acids longer than CXCR3-A^7^. CXCR3-B, mainly found in endothelial cells, displays angiostatic functions^7, 8^. Furthermore, CXCR3-B also has anti-proliferative and apoptotic functions^9–12^. In stark contrast, CXCR3-A activation induces proliferation, migration and cell survival, notably in tumor cells^13–15^. Little is known about the role of the third isoform, CXCR3-alt which is only expressed at low levels *in vivo* ^16, 17^.

CXCR3 ligand stimulation activates the MAPK/ERK and the PI3 kinase/AKT pathways, and induces chemotactic effects^16, 17^. For the activation of the different signaling pathways, specific G-proteins are recruited^18–20^. Since CXCR3 isoforms exhibit opposite biological functions, studies have been carried out to determine which G-proteins are recruited following receptor stimulation. CXCR3-A binds Gα-i/o or Gα-q proteins which activate pro-migratory and proliferative pathways including PI3K/AKT or MAPK (ERK, JNK)^13, 21^. On the other hand, CXCR3-B recruits Gα-s protein which inhibits cell proliferation and migration via adenylate cyclase activation and cAMP generation. Indeed, cAMP stimulates protein kinase A (PKA) which inhibits proliferation and activates apoptosis in endothelial cells^11^.

In tumor cells, expression of CXCR3, and more specifically of CXCR3-A, has been correlated with poor prognosis in patients with breast cancer, colon cancer, glioma and osteosarcoma^22–25^. CXCR3-B expression has been found to correlate with favorable prognosis in kidney and gastric cancers^9, 10^. Thus, activation of CXCR3 isoforms leads to distinct biological behaviors and clinical outcomes. This could possibly be explained by very early changes in the signaling pathway such as modifications of receptor conformation in response to ligand activation. Another issue that remains to be exploited is the specific inhibition of CXCR3-A, which has a pro-tumor activity, without affecting the CXCR3-B isoform. This could be clinically highly relevant.

In this article, we studied CXCR3-A and CXCR3-B ligand-induced conformational changes by plasmon waveguide resonance (PWR) and subsequent receptor activation by calcium flux analysis. Differences in conformational changes for CXCR3-A and CXCR3-B after ligand binding were observed, leading to opposite signaling, which may explain the different biological responses triggered by the two receptor isoforms. Titration experiments with SCH546738, an antagonist of CXCR3, helped to define the critical concentration which strongly inhibited CXCR3-A but not CXCR3-B activation.

## Results

### Specific CXCR3 isoform overexpression

To investigate the dual role of the two CXCR3 isoforms, Hek-293 cells were stably transfected with plasmids encoding CXCR3-A (HEK-CXCR3-A) or CXCR3-B (HEK-CXCR3-B), both fused to GFP. CXCR3 overexpression was assessed by qPCR (Fig. S1A). Overexpression of CXCR3-A had no effect on endogenous CXCR3-B expression and *vice versa* (Fig. S1A). As a control, HEK-293 cells transfected with GFP empty vector (HEK-CTRL) were also established and immunofluorescence staining was performed in these three cell lines (Fig. S1B). In non-stimulated condition, both CXCR3-A and CXCR3-B were localized in secretory vesicles near to the nucleus. SiRNA for human CXCR3-A and CXCR3-B were transfected in HEK-CXCR3-A and HEK-CXCR3-B cells and used to verify CXCR3 overexpression (Fig. S1C and D). SiRNA CXCR3-9 led to an almost complete downregulation of CXCR3-A or CXCR3-B overexpressed in cells as evidenced by immunofluorescence and western-blot.

### Agonist binding and cell-mediated signaling of CXCR3-A versus CXCR3-B receptor

**Figure 1:**
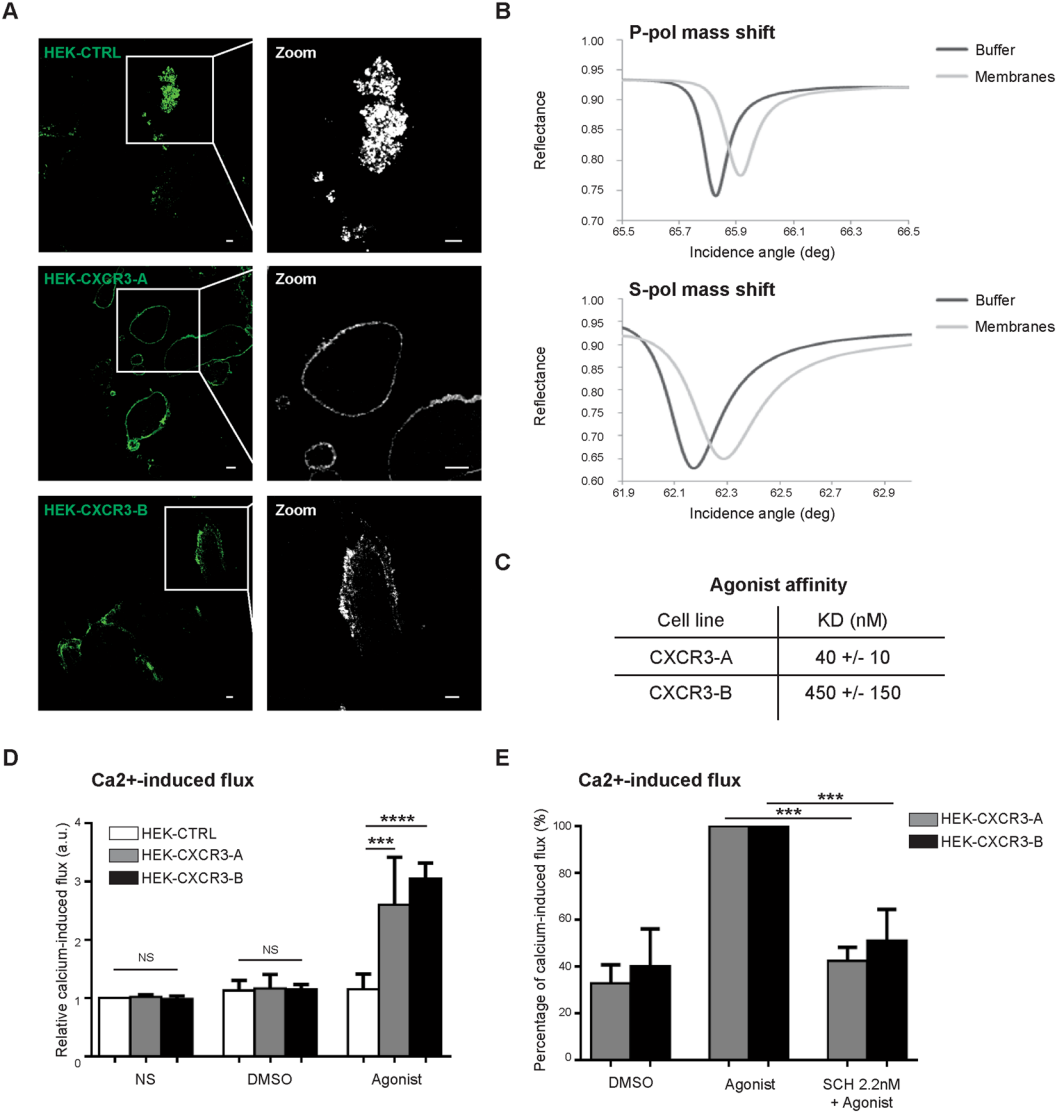
Conformational change and CXCR3 activation in response to agonist stimulation. (**A**) Immobilization of HEK-CTRL, HEK-CXCR3-A and HEK-CXCR3-B cell fragments on glass slides. Sections were observed at 630× magnification under a confocal laser-scanning microscope (Nikon eclipse Ti) and images were acquired with the NIS Image browser software. Scale: 5 µm. (**B**) PWR spectra for the PWR sensor before and after immobilization of the cell membrane fragments obtained with p- and s-polarized light. (**C**) K_D_ values for PS372424 interaction with CXCR3-A in HEK-CXCR3-A and HEK-CXCR3-B cells. (**D**) Relative calcium-induced flux in HEK-CTRL, HEK-CXCR3-A and HEK-CXCR3-B cells after 20 seconds of PS372424 stimulation. a.u.: arbitrary unit. (**E**) Relative calcium flux in non-stimulated (DMSO) and stimulated HEK-CXCR3-A and HEK-CXCR3-B cells, pretreated or not with CXCR3 antagonist SCH546738. DMSO: negative control. All the results represent three independent experiments combined to calculate mean and SEM, and values were normalized to those obtained for the control cells. ***P < 0.001.

Ligand-mediated activation of CXCR3 was studied by PWR and flow cytometry measuring calcium flux. The former technique allows to determine the affinity, ligand-binding kinetics and anisotropy of the events occurring at the sensor surface^26^. This technique gives the possibility of following resonance with two types of polarizations, *p*- and *s*-polarizations. PWR can be applied both to artificial lipid model systems with reconstituted receptors or to cell fragments^27–34^. In the present study, cell membrane fragments from cells expressing the CXCR3-A and CXCR3-B receptors were immobilized on the PWR sensor which is composed of a prism coated with a silver layer and overcoated with a silica layer. The prism is pre-treated with polylysine to promote cell adhesion by electrostatic interactions between the negative charges provided by the cell surface glycosaminoglycans (GAGs) and the positive charges of polylysine. The quality of HEK-CTRL, HEK-CXCR3-A and HEK-CXCR3-B membrane fragments was verified by confocal microscopy by visualizing the GFP-tagged receptors using glass slides of the the same material (silica) than the outer layer of the sensor surface (Fig. 1A). The presence of fluorescent membrane fragments on the glass slide of few μm were evidenced in agreement with reported literature^35^.

The PWR spectra showed a large increase in the resonance minimum position (about 100 mdeg) for both polarizations, which correlated with mass increase due to capture of the cell fragments (Fig. 1B). The *p*-polarization corresponds to the plane perpendicular to the sensor surface and the *s*-polarization to the plane parallel to the sensor surface. Spectral shifts were larger for the *p*- than for the *s*-polarization (Fig. 1B). The greater resonance shift observed for *p*- than *s*-polarization indicates that lipids and proteins are oriented with their long axis perpendicular to the sensor surface. Considering that the immobilization occurs by electrostatic interactions between polylysine on the sensor and GAGs on the cell surface, such molecular orientation is expected (Fig. S2). Agonist (PS372424) affinity for both CXCR3 isoforms was investigated by adding increasing concentrations of the CXCR3 agonist until saturation. At the same time, we followed the PWR spectral changes that reflect ligand-induced receptor conformational changes upon formation of the receptor/ligand (R/L) complex. The dissociation constant was found to be different for CXCR3-A and CXCR3-B by a magnitude of 10-fold (CXCR3-A K_D_: 40 +/− 10 nM, CXCR3-B K_D_: 450 +/− 150 nM) (Fig. 1C; Fig. S3). This is the first time﻿, as far as we know, that ligand binding affinities have been separately determined for the two receptor isoforms. Reported literature mention CXCR3 without specifying whether the two isoforms are presented and, if it is the case, in which ratio. It is thus not possible to perform a proper comparison between the values determined herein and the literature. Nonetheless, an IC_50_ of 42 nM has been reported for PS372424 binding^36^ which is rather is good agreement with the value of 40 nM that we have determined for the binding to CXCR3-A. Regarding SCH546738, its affinity to CXCR3 has been reported to be 0.4 nM or to vary from 0.8–2.2 nM^37, 38^. The dissociation constants that we have obtained are of 0.4 nM for CXCR3-A and 1- 0.5 nM for CXCR3-B and thus are in the same range as the reported ones. Receptor activation was assessed by measuring calcium flux after GPCR activation (Fig. 1D and E). Both CXCR3 isoforms were able to increase intracellular calcium flux after agonist stimulation (Fig. 1D). Thus, despite different receptor affinities, the CXCR3 agonist was able to activate both, CXCR3-A and CXCR3-B. When cells were pretreated with SCH546738, a pharmacological CXCR3 inhibitor, both receptors were no longer activated and no change in intracellular calcium flux was noticed (Fig. 1E).

### Conformational change of CXCR3 after agonist binding

**Figure 2:**
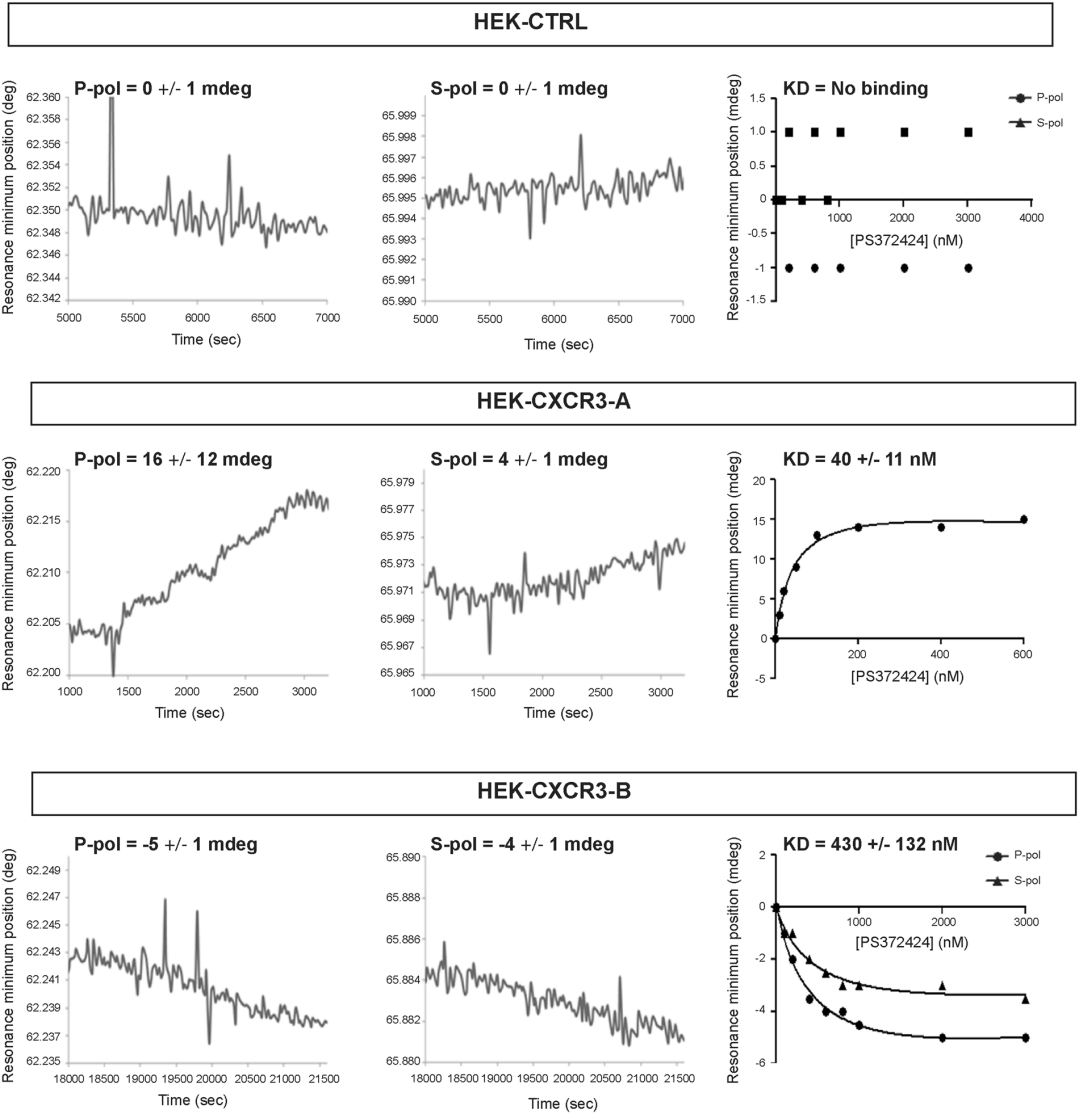
Time-dependent conformational change and ligand affinity. Changes in the minimum resonance position following incremental addition of CXCR3 agonist PS372424 to HEK-CTRL, HEK-CXCR3-A and HEK-CXCR3-B cell fragments as a function of time obtained with *p*-(left panel) and *s*-(right panel) polarized lights. Binding curve and K_D_ values for PS372424 interaction with CXCR3-A and CXCR3-B were indicated in each condition. All the results represent three independent experiments combined to calculate mean and SEM, and values were normalized to those obtained for the control cells.

Membrane fragments from HEK-CTRL cells, which were not transfected with the vector construct encoding CXCR3, did not show ligand binding as no changes in either polarizations were seen after agonist addition. In contrast, membrane fragments of cells expressing either CXCR3-A or CXCR3-B responded to ligand stimulation in a saturable manner (Fig. 2; Fig. S3). This indicates that, at the concentrations tested, only CXCR3 receptor-containing membrane fragments responded to ligand stimulation. PWR is sensitive to both, mass and structural changes. Inasmuch as the ligand is far too small to contribute to a mass change by itself, the PWR spectral changes observed correspond to ligand-induced receptor conformational changes (upon formation of the R/L complex). From the saturable and hyperbolic binding response, a dissociation constant was determined. The cell fragments are quite stable after sensor immobilization as no alterations in the spectra were observed after repetitive and extensive washing with buffer. Furthermore, lipids and receptors are oriented with their long axis along the p-polarization axis as shown by the anisotropic response. Thus, spectral modifications observed in *p*-polarization reflect changes along the receptor main axis, while modifications in *s*-polarization reflect changes along the receptor minor axis (Fig. S2). In HEK-CXCR3-A cells, agonist binding led to an increase in the resonance minimum position for both *p*- and *s*-polarization. A positive shift in the resonance angle is correlated with an increase in the refractive index due to a mass increase in the system. Since the ligand, by itself, is too small to be able to produce such spectral changes, the response can only be attributed to conformational change in the receptor which is reflected by an increase in mass density. Considering that the response is anisotropic (the shift in *p*-polarization was much higher than in *s*-polarization), this indicates that ligand-induced receptor conformational change reflects receptor elongation and increases in packing and ordering of the transmembrane (TM) helices (Fig. 2). Strikingly, CXCR3-B ligand activation resulted in an opposite response with negative shifts for both, *p*- and *s*-polarization (Fig. 2; Fig. S3). The negative shift, while of considerably smaller magnitude, results from a slight decrease in the mass density in the system and can be attributed to a conformational change leading to a looser and less ordered arrangement of the TM helices accompanied by a slight decrease in length. Thus, agonist-mediated CXCR3 activation leads to opposite conformational changes depending on the CXCR3 isoform.

### CXCR3 antagonist induces conformational changes in CXCR3 which prevent agonist binding

**Figure 3:**
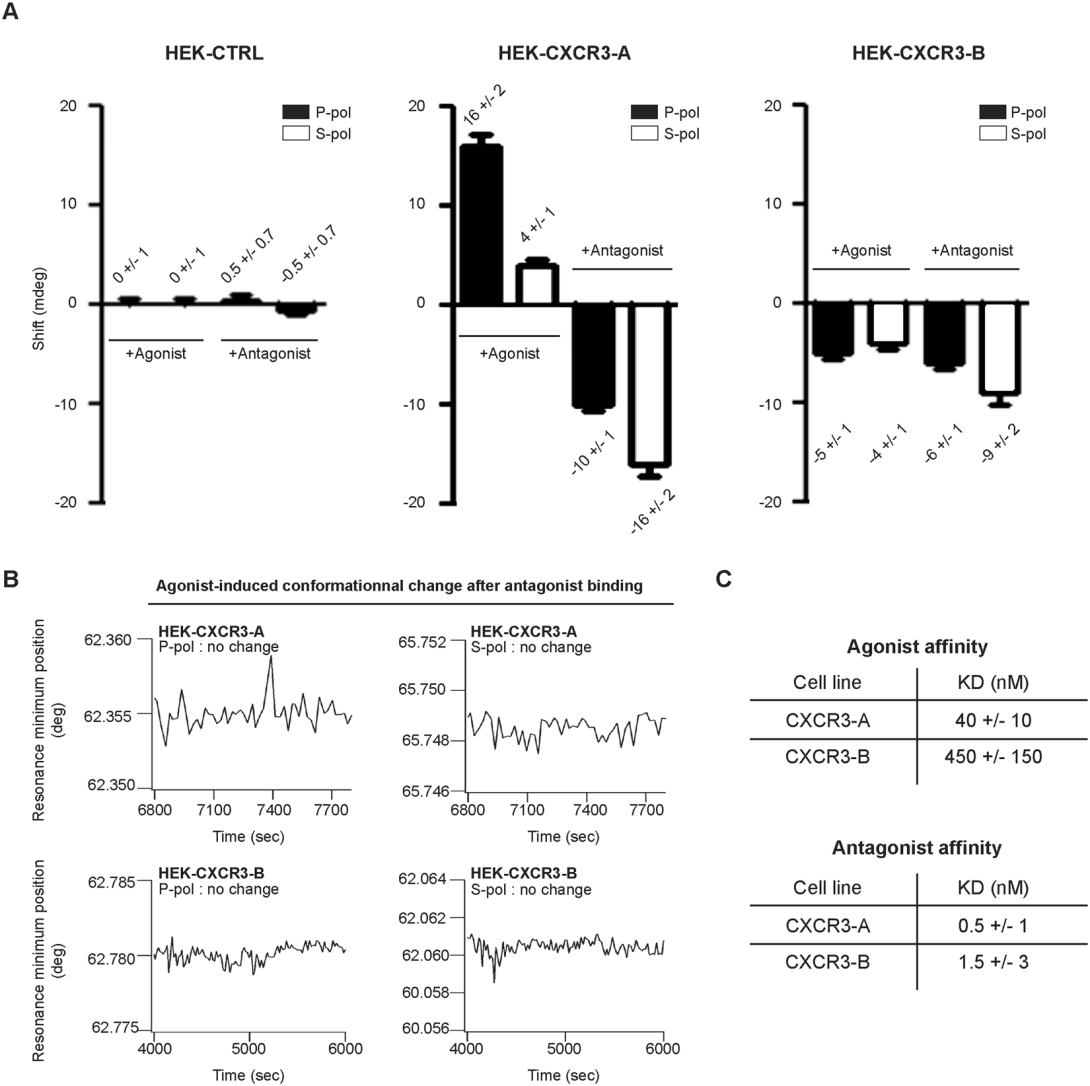
Conformational change in response to antagonist. (**A**) Changes in the minimum resonance position following incremental addition of CXCR3 agonist PS372424, or CXCR3 antagonist SCH546738 to HEK-CTRL, HEK-CXCR3-A and HEK-CXCR3-B cell fragments as a function of time obtained with *p*-(left panel) and *s*-(right panel) polarized lights. (**B**) PWR shifts obtained with *p*-(left panel) and *s*-(right panel) polarized lights for HEK-CXCR3-A and HEK-CXCR3-B after treatment of SCH546738 (1 h at 2.2 nM). (**C**) KD values for PS372424 or SCH546738 interaction with CXCR3-A in HEK-CXCR3-A and HEK-CXCR3-B cells. All the results represent three independent experiments combined to calculate mean and SEM, and values were normalized to those obtained for the control cells.

We next conducted PWR experiments with the CXCR3 antagonist SCH546738. Like with the agonist, cell fragments of HEK-CTRL did not respond to the antagonist indicating absence of non-specific ligand binding to other cell components than the receptor (Fig. 3A). We showed that both, CXCR3-A and CXCR3-B could bind SCH546738 (Fig. 3A) but this resulted in different receptor conformational states than those observed for the agonist. Binding of SCH546738 to HEK-CXCR3-A cells, resulted in negative shifts for both *p*- and *s*-polarization which is in contrast to the agonist response described in the previous section (Fig. 3A). For HEK-CXCR3-B cells, negative shifts for both polarizations were observed for SCH546738, similar to the agonist (Fig. 3A). However, the responses for CXCR3-B was still different to that of the agonist, both in terms of magnitude (related to the amplitude of the receptor conformational change) and anisotropy (ratio between the shifts in the two polarizations). The affinity of SCH546738 for both CXCR3-A and CXCR3-B was found to be high with K_D_ values of 0.5 nM and 1.5 nM respectively (3-fold lower K_D_ for CXCR3-A than for CXCR3-B). At saturating SCH546738 concentrations (2.2 nM, above the K_D_ values), agonist binding to both HEK-CXCR3-A and CXCR3-B was completely abolished (Fig. 3B).

### Low SCH546738 concentrations predominantly inhibit CXCR3-A activation

**Figure 4:**
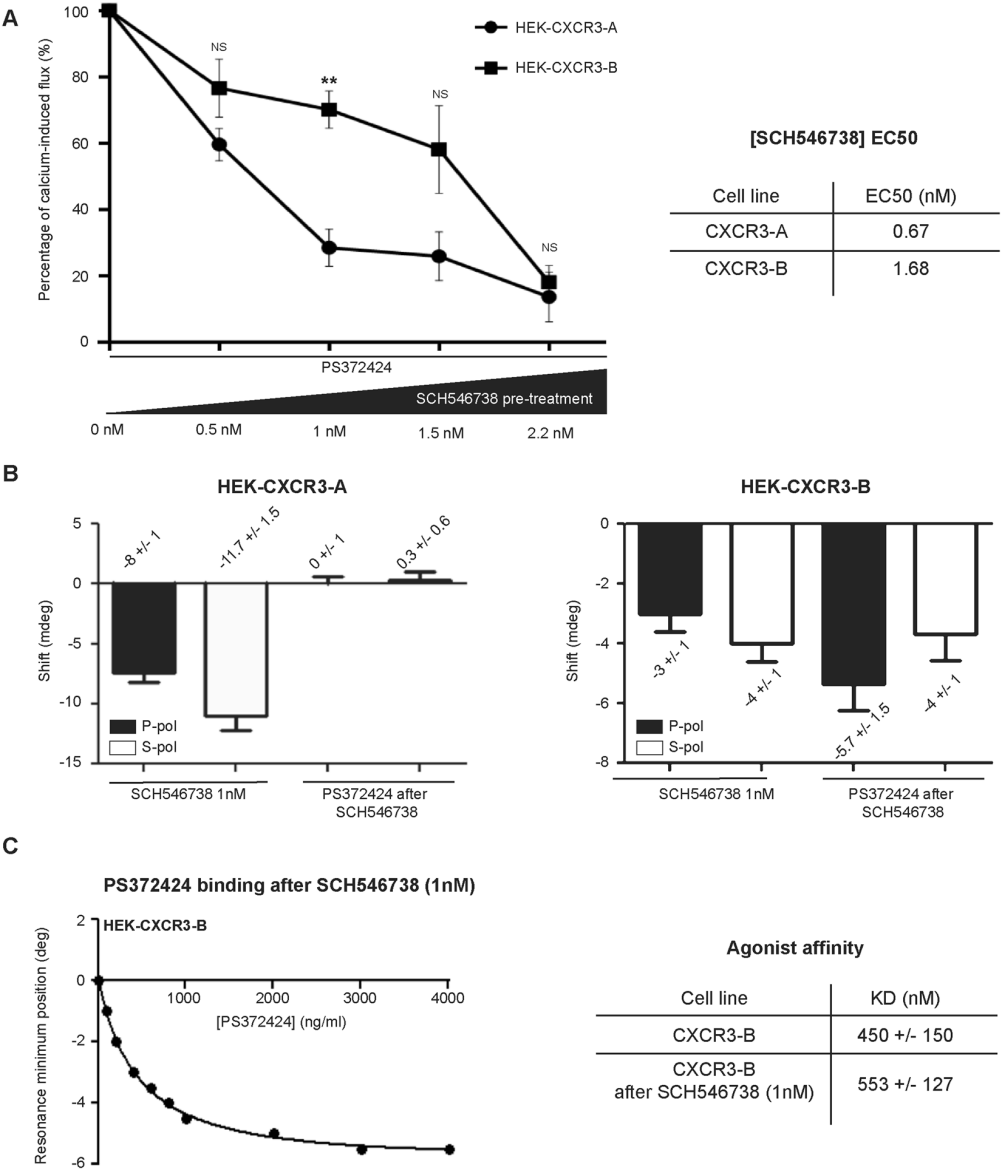
Calcium-flux and PWR shifts in the presence of agonist and antagonist. (**A**) Relative calcium-induced flux in HEK-CXCR3-A and HEK-CXCR3-B cells after 1 h of SCH546738 pre-treatment at concentration from 0 to 2.2 nM. SCH546738 concentration inhibiting 50% of the relative calcium-induced flux were indicated as EC_50_ values. (**B**) PWR shifts obtained with *p*-(left panel) and *s*-(right panel) polarized lights for HEK-CXCR3-A (left panel) and HEK-CXCR3-B (right panel) after treatment of SCH546738 alone (1 h at 1 nM) or after addition of PS372424 after SCH546738 at 1 nM. (**C**) Binding curve and K_D_ values for PS372424 interaction with CXCR3-B before and after SCH546738 treatment at 1 nM. All the results represent three independent experiments combined to calculate mean and SEM, and values were normalized to those obtained for the control cells.

Since SCH546738 has 3-fold higher affinity for CXCR3-A than for CXCR3-B, we sought to determine the concentration of SCH546738 which would only inhibit CXCR3-A without affecting CXCR3-B. We showed that low concentrations of SCH546738 greatly inhibited CXCR3-A activation as measured by calcium flux, the effect on CXCR3-B activity being lower in this case (Fig. 4A). More precisely, 1 nM of SCH456738 inhibited more than 70% of CXCR3-A activation and only 25% of CXCR3-B activation. The EC_50_ values for SCH546738 for CXCR3-A and CXCR3-B were 0.67 nM and 1.68 nM respectively (Fig. 4A). We next performed PWR experiments to follow the agonist-induced conformational change after antagonist (1 nM of SCH546738) treatment. We demonstrated that SCH546738 at 1 nM totally abrogated agonist binding to CXCR3-A (Fig. 4B). This was different in the case of CXCR3-B because binding was still observed in this case. Thus, 1 nM of SCH546738 inhibited CXCR3-A activation, but not significantly CXCR3-B activation. The affinity of the agonist for CXCR3-B before and after treatment with SCH546738 (1 nM) was not changed (450 +/− 150 nM and 553 +/− 127 nM, respectively) (Fig. 4C).

## Discussion

Chemokine receptor CXCR3 is classically involved in the chemotactic activity of the IFN-γ-induced CXC chemokines on activated T lymphocytes and NK cells^39^. CXCR3 interacts with CXCL9-11, CXCL4 and CXCL4L1. CXCR3 seems also to be involved in neurodegeneration (e.g. multiple sclerosis), inflammation, autoimmune disease (e.g. autoimmune thyroiditis) malaria infection, cancer, atherosclerosis and neoangiogenesis^1–6, 40^. CXCR3 is express in various cell types including endothelial cells, immune cells or tumor cells. In smooth muscle cells, CXCR3 is particularly expressed in areas associated with atheromatous plaque, making its expression a risk factor of atherosclerosis^41^. Although CXCR3 is recognized as the functional receptor for angiostatic chemokines, the presence of 3 spliced variants including CXCR3-A, CXCR3-B and CXCR3-alt complicates the understanding of its biological function.

It has been reported that the angiostatic and anti-proliferative effects of CXCR3 are carried by the CXCR3-B isoform, which is mainly expressed in endothelial cells^7, 8^. In contrast, CXCR3-A activation induces proliferation, migration, cell survival and has been characterized as a pro-tumor receptor^13, 14^ in pancreatic, breast, renal and prostate carcinoma as well as in glioma cells^13, 14, 21, 42–44^. However, CXCR3-B expression has also been reported in several tumor types including prostate carcinoma, renal cell carcinoma and the biological effect is generally inhibitory^9, 10, 13^. This raises the question which and how receptors are activated preferentially in this case. CXCL4L1 and CXCL10 are able to activate tumor cell-bound CXCR3-A, while CXCL9 and CXCL11 mainly act via the immune system^45, 46^ CXCR3-B, as previously mentioned, seems to be present in the tumor vasculature and has mainly an inhibitory effect.

To gain insights into the activation mechanisms of the two CXCR3 isoforms, CXCR3-A and CXCR3-B were overexpressed in HEK-293 cells. We used PWR to investigate both the affinity and the receptor conformational changes of both isoforms in response to agonist and antagonist stimulation. CXCR3-A and CXCR3-B both bind the agonist and are activated as evidenced by calcium-induced flux analyses. The agonist exhibited a stronger affinity for CXCR3-A than CXCR3-B. An IC_50_ value of 42 nM has been reported for PS372424/CXCR3 binding^36^ which is rather in good agreement with the value of 40 nM we have determined in our study. However, this latter study does not distinguish between the two isoforms. Upon agonist exposure, CXCR3-A is preferentially activated especially when lower agonist concentrations are used. Agonist activation resulted in totally different receptor conformational states for CXCR3-A when compared to CXCR3-B. This difference may be linked to the pro-tumor effect of CXCR3-A.

It is known that G protein recruitment by the two CXCR3 isoforms is different and leads to signaling cascades resulting in opposite biological effects. CXCR3-B activation induces Gα-s protein recruitment, cAMP formation and stimulation of protein kinase A (PKA). This inhibits endothelial cell proliferation and activate apoptosis via the MAPK/P38 pathway^11, 12^. On the contrary, CXCR3-A activation leads to Gα-i/o or Gα-q proteins binding which favors a pro-tumor response (through the activation of these pathways: Phospholipase C, PI3K/AKT, MAPK/ERK, MAPK/JNK). This leads to cell proliferation, migration and invasion^13, 21^. In our study, we used PWR to assess CXCR3-A and CXCR3-B conformational dynamics after agonist stimulation. PWR is a validated method to investigate, besides ligand affinity, receptor dynamics after agonist or antagonist exposure^27–34^. PWR studies revealed that, in addition to differences in affinity, PS373434 also induces different conformational changes depending on the CXCR3 isoforms. In the case of CXCR3-A, the conformational change consisted in an increase in receptor packing and ordering accompanied by elongation. On the contrary, a decrease in packing and ordering of the receptor was seen for CXCR3-B. This is the first time that the dynamics of receptor conformation of CXCR3 isoforms is studied in a comparative manner. Distinct conformational states may be the result of different spatial arrangements of the TM helices which can be accompanied by alterations in the organization of the surrounding lipid membrane. The reason for these totally opposing behaviors of the two CXCR3 isoforms in response to ligand stimulation is not known. One can only speculate that the different amino-terminal domains for CXCR3 isoform are involved. Indeed, CXCR3-B has a 52 amino-acid extension at the N-terminus which is not found in CXCR3-A^7^. This extension may affect the spatial organization of CXCR3-B after agonist binding. The specific reasons are not known at present. However, it has been shown that CXCR3-A and CXCR3-B are sulfated on its N terminus and that sulfation is required for binding and activation by CXCL9, CXCL10 and CXCL11. Furthermore, deleting the proximal 16 amino acid residues of the N-terminus led to a decrease in CXCL10 and CXCL11 binding and receptor activation but did not modify CXCL9 binding or CXCL9-dependent activation^47^. Two other potential sulfation residues are found specifically in the 52 amino acid extension of CXCR3-B at amino acid position Y6 and Y40. These residues may possibly affect the interaction of the ligand with CXCR3-B and have a specific effect on receptor activation.

Another possibility might be related to the different lipid environment that surrounds the two receptor isoforms. This could arise from the different partitioning of receptors in lipid domains (ordered/disordered) at the level of the plasma membrane^48^. The lipid environment could have an impact on receptor activation and signaling as it has been demonstrated for other receptor systems by PWR and other approaches^29, 49^. It is possible that the N-terminal sequence in CXCR3-B will influence the interaction of the receptor with the surrounding lipids differently to CXCR3-A. The precise reasons of how this is occurring are not known and will require detailed molecular studies.

The distinct behavior of both receptor isoforms and the different conformational states adopted could contribute to the recruitment of a different set of G-protein, and, thus, lead to the activation of different signaling pathways. The precise mechanisms of how the conformational changes will impact on G-protein recruitment are also not yet understood and will be the focus of future investigations.

It has been proposed that CXCR3-A and CXCR3-B constitutes a balance for tumor progression and represent “Stop and Go “signals^13^. If the expression is in favor of CXCR3-A, pro-tumor pathways related to migration and invasion are activated (“Go” signal of tumor progression). On the contrary, if the balance is in favor of CXCR3-B, migration and tumor invasion are inhibited and the “Stop” signal is activated^13^. Thus, the relative expression between the two CXCR3 isoforms in tumor cells determines the behavior of cells in response to agonist stimulation. Since CXCR3 agonists have a higher affinity for CXCR3-A than for CXCR3-B, CXCR3-A is the predominant receptor when cells are expressing both isoforms. Furthermore, CXCR3-A is mostly expressed at higher levels than CXCR3-B as it is occurring in tumor cells^14^.

In fact, under non-stimulated conditions, the pattern of CXCR3-B localization is more diffuse with fewer cells with CXCR3-B presents at the cell membrane, as detected by immunofluorescence microscopy. The majority of CXCR3-B remains in the cytoplasm. Despite the localization at the plasma membrane (Fig. S1C), CXCR3-A is also significantly localized in the intracellular compartment (Fig. S1B) similar to CXCR3-B. Our results are in agreement with the observation reported in breast cancer cell lines^24, 42^ which showed that under non-permeabilized conditions two populations of cells were detected: cells expressing CXCR3 on their surface and cells without any surface expression. After permeabilization, most of the cells stained positive for CXCR3. In our study, CXCR3-A and CXCR3-B both relocalizes at the cell membrane upon agonist stimulation, (Fig. S4). Thus, cell surface exposure of the two CXCR3 isoforms is regulated by chemokine stimulation. CXCR3-A and CXCR3-B are possibly localized and recycled in the same intracellular compartments and the differences in the cellular responses are principally due to the recruitment of different signalling modules.

However, we cannot formally exclude the possibility that the synthesis or recycling compartments are different to some extent for both receptor isoforms and that the lipid composition might play a role. Indeed, the lipid composition of the plasma membrane may differ from that of intracellular organelles. For example, vesicles from the Golgi apparatus contain different distribution of sphingolipids (such as sphingomyelin and ceramides). Additionally, intracytoplasmic CXCR3 may also be derived from endosomes, their lipid composition reflecting that of the plasma membrane. Furthermore, results from our laboratory indicate that CXCR3-A is clathrin-dependent and caveolin independent (Nature Communications, in press), which may not be the case for CXCR3-B. The differences in membrane localization between CXCR3-A and CXCR3-B may be due to some divergence in the intracellular trafficking between both receptor isoforms.

Since no specific CXCR3-A inhibitors exist, we decided to investigate whether CXCR3-A or CXCR3-B could be preferentially inhibited by varying antagonist concentrations. We used SCH546738, a potent and specific CXCR3 inhibitor which blocks the biological activity *in vitro* and *in vivo* of both receptor isoforms^38^. The dissociation constants obtained by PWR are of 0.4 nM for CXCR3-A and of 1.5 nM for CXCR3-B. This is similar to affinities determined in other reports and by other methods (from 0.4–2.2 nM^37, 38^. We demonstrated that the affinity of the antagonist for CXCR3-A is 3-fold greater than that for CXCR3-B. We incubated HEK-CXCR3-A or HEK-CXCR3-B cells with increasing SCH546738 concentrations and showed that low SCH546738 concentrations efficiently inhibited CXCR3-A. At 1 nM, the antagonist is bound preferentially to CXCR3-A, but only weakly to CXCR3-B which can still be activated by the ligand. Since CXCR3-A represent the “pro-tumor receptor” this means that, in a tumor context, SCH546738 will preferentially inhibit tumor progression when lower concentrations are used.

**Figure 5:**
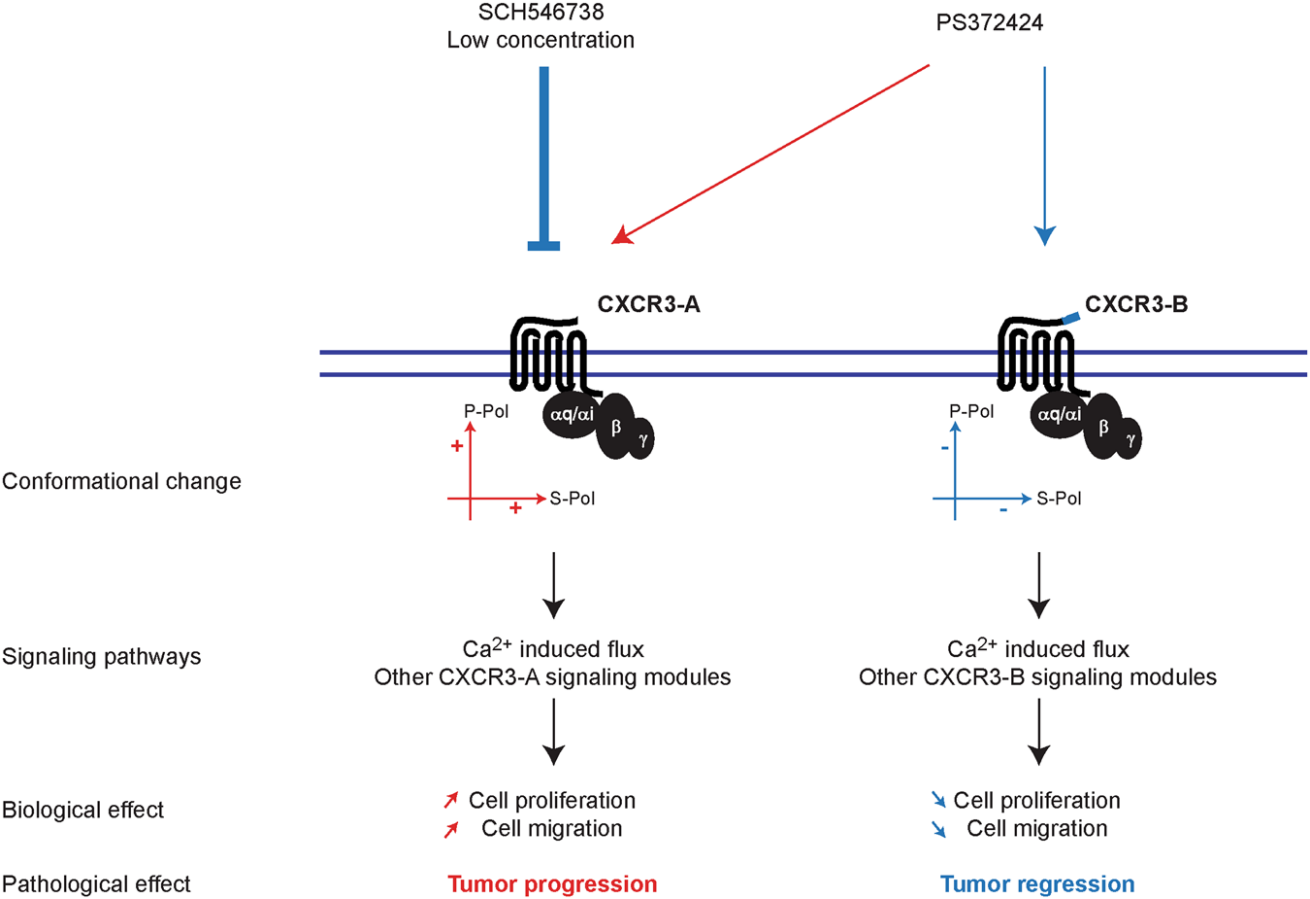
Model describing the differences in CXCR3 isoform activation and function.

Taken together, our results demonstrate that CXCR3-A and CXCR3-B have distinct behavior when stimulated with an agonist and undergo different conformational changes (Fig. 5). Furthermore, we demonstrate that ligand affinities are different for both receptors, which is in agreement with already published data^7, 15, 17, 50^. Finally, preferential CXCR3-A receptor blockade can be achieved by using low concentrations of specific inhibitors. This has consequences for anti-tumor therapy when CXCR3 blocking agents are used considering that CXCR3-A has a pro-tumor properties and CXCR3-B exhibits anti-tumor activity.

## Materials and Methods

### Construction of CXCR3 expression plasmids

The coding regions of human CXCR3-A or CXCR3-B cDNA were digested with BamHI and KpnI restriction enzyme and inserted into the pEGFP-N2 vector (BioSignal Packard). These vectors were a kind gift from Dr. Alexandre Dubrac (Yale University).

### Cell culture

Human Embryonic Kidney (HEK-293) cell lines, were regularly tested for contamination and were all mycoplasma-free. Cells were cultured in Dulbecco’s Modified Eagle’s medium (DMEM, Thermo Fisher Scientific) supplemented with 10% Fetal Bovine Serum (FBS), 5% antibiotics (Penicillin and Streptavidin) and 5% L-glutamine. All cells were grown at 37 °C, 5% CO_2_ and split at 70–90% of confluence with 0.25% Trypsin. Cell transfection was performed using Effecten kit (Qiagen) according to the manufacter’s instruction. HEK-CTRL (HEK-293 stably transfected with empty pEGFPN2 vectors) HEK-CXCR3-A cells and HEK-CXCR3-B (HEK-293 stably transfected with pEGFPN2 + CXCR3-A or pEGFPN2 + CXCR3-B vectors, respectivly) were grown in the presence of Zeocin (200 µg/ml). For inhibition experiments, cells were pretreated 1 h with the CXCR3 antagonist SCH546738 at dose-dependant concentration. SCH546738 is a small molecular lipophilic molecule, with the chemical formula: C_23_H_31_Cl_2_N_7_O. Its affinity to CXCR3 has been reported to vary from 0.4–2.2 nM^37, 38^. For this reason a concentration range from 0.5 to 2.2 nM has been used.

### Gene expression analysis

Total RNA was isolated from cells using Trizol reagent (Invitrogen) according to the manufacter’s instruction. 1 µg of total RNA was reverse-transcribed into cDNA using the high-capacity cDNA reverse transcription kit (AbCam 4368814). The resulting cDNAs were amplified using specific primers for the genes of interest. GAPDH (glyceraldehyde-3-phosphate dehydrogenase), and HPRT (hypoxanthine guanine phosphoribosyl transferase, Fisher Scientist, Hs99999909_m1) were used as internal controls. We applied the real-time PCR method using the FG POWER SYBR GREEN PCR (Fisher Scientist, 10658255) for CXCR3-A: forward sequence CCATGGTCCTTGAGGTGAGTG and reverse sequence AGCTGAAGTTCTCCAGGAGGG. TAQMAN UNIV PCR MASTER MIX (Fisher 10157154) was used for CXCR3-B: probe sequence (6FAM)CCCGTTCCCGCCCTCACAGG, forward sequence TGCCAGGCCTTTACACAGC and reverse sequence TCGGCGTCATTTAGCACTTG. Real-time PCR reactions were performed on a StepOnePlus Real-Time PCR system using StepOne software V2.3 (Applied Biosystems).

### Small interfering RNA knockdown experiments

For inhibition of CXCR3 expression, cells were transiently transfected with annealed siRNA. CXCR3 specific siRNAs were used (ON-TARGETplus Human CXCR3 (2833). A siRNA-random (siRNA CTRL, SR-CL000-005) was used as a negative control. Transfection was performed using lipofectamine RNAimax kit (Fisher 10601435) in accordance with the manufacturer’s protocol, with siRNA at a final concentration of 20 nM in media without antibiotics for 6 hours at 37 °C. After transfection, cells were washed with PBS and fresh complete media was added for 48 h.

### Plasmon waveguide resonance (PWR)

PWR measurements were performed in a homemade instrument equiped with a He-Ne laser, with a fixed wavelenght in the visible region (λ = 632 nm; Melles Griot), a rotating table allowing the incident angle to be changed by steps ≤1 mdeg (thus resolution being on that order; Newport) and a photodiode detector (Hamamatsu) to measure the reflected light as a function of the incident angle. The polarization angle of the incident beam is placed at 45° allowing both *p*- and *s*-polarized spectra to be obtained within the same angular scan. The sensor consisted in a BK-7 prism coated with silver and silica (to support waveguide modes)^36^. All measurements were performed at 22 °C in a temperature controlled room. More details about this technology can be found^26^. The protocol for adhesion of cell fragments on silica (glass slides or PWR sensor) was adapted from reported work from Vogel and collaborators^35^. To be mentioned that the PWR sensor cannot be directly used for confocal microsocpy due to the presence of the silver layer that reflects light. Therefore glass slides were used instead that perfectly represent the last layer of the PWR sensor (a thick silica layer). Briefly, the silica surface was washed with Ethanol and cleaned and activated by Plasma cleaner for 2 min (Diener). The silica surfaces are then incubated with a solution of polylysine (0.1 mg/mL) for 40 minutes following wash with PBS buffer. Cells grown to less than 50% confluence are washed with PBS and covered with water to induce osmotic swelling of the cells. Imediately, the glass coverslip of the sensor is placed directly on top of cells. Pressure is applied for about 2 min on the glas slide or prism to induce cell rupture and caption of cell fragments. After that they are removed ripping off cell fragments containing specially the upper membrane. The glass slide or sensor is washed with PBS to remove cell debris and kept with buffer to prevent drying and loss of membrane protein activity. PWR measurements are performed right away. The PWR cell sample (a teflon block with a volume capacity of 250 μL) is placed in contact with the prism and filled with PBS. After cell fragment deposition there are positive shifts in the resonance minimum position that are correlated with the total mass gain occurring. We have observed spectral shifts that correlate with those observed for the deposition of lipid model membrane. In the case presented here, there are areas of the sensor covered with cell membranes and others uncovered, the PWR signal takes into account both covered and uncovered areas as the laser spot makes about 0.5 mm in diameter. At same time, covered areas include both lipids and proteins, so possess a higher mass than that of a pure lipid membrane. Following stabilisation of the signal (no changes in the resonance minimum position with time), the ligand is added in a incremental fashion to this chamber and specral shifts followed with time. Ligand affinity to the receptor present in the cell membrane fragments is calculated by ploting the shifts in the resonance minimum position as a function of ligand concentration and fitting using an hyperbolic function that describes ligand binding to a protein (single site) (Graph Pad Prism).

### Western-blot

Cells were washed twice with phosphate-buffered saline (PBS) and dissolved in lysis buffer (10 mM Tris-HCl, pH 7.4, 150 mM NaCl, 0.5% NP-40, 1%TritonX-100, 1 mM EDTA) supplemented with protease and phosphatase inhibitors cocktails (Roche). Protein concentration was quantified by Bradford assay (Euromedex). 50 µg of cell lysates were resuspended in Laemmli Buffer (62.5 mM Tris pH6.8, 10% glycerol, 2.5% SDS, 2.5% β-mercapto-ethanol) and boiled for 5 minutes. Samples were subjected to electrophoresis and transferred. Proteins were electroblotted onto a polyvinylidene difluoride (PVDF) membrane (Fisher Scientist, 10344661). Membranes were incubated with blocking buffer (EuroBio 927-40003) for 1 h, probed overnight at 4 °C with the primary antibodies of interest, followed by detection using secondary antibodies coupled to Fluoroprobes (goat anti mouse IR Dye 680CW, 926-68020; goat anti mouse IR Dye 800CW) and Odyssey infrared imaging system (Li-Cor Biosciences, Nebraska, US). Densitometry analysis was performed using Image Studio Lite 4.0 software.

### Immunofluorescence staining

Cells were seeded on glass coverslips and fixed 10 minutes with 4% paraformaldehyde at room temperature. Cells were permeabilized 10 minutes with 0.1% TritonX-100, washed with PBS and incubated for 1 h with blocking buffer (PBS containing 2% FBS and 1% BSA) at room temperature. Cells were incubated with primary antibodies diluted in blocking buffer overnight at 4 °C, washed with PBS, and incubated with secondary antibodies diluted in blocking buffer (Interchim, FluoProbes® 488 H, 547 H or Goat or Donkey Anti-Mouse or Rabbit IgG (H + L)). Dapi was used as counterstained agent to label nuclei (Fisher Scientist, 10374168).

### Confocal microscopy

Confocal images were acquired with a Nikon Eclipse Ti inverted laser scanning fluorescence microscope equipped with acquisition software (NIS-Element, NIS) and ×63 oil immersion objective (numerical aperture [NA], 1.4). Each channel was imaged sequentially before merging.

### Calcium measurement

Intracellular calcium concentration was measured using Calcium Assay Kit (BD 640176) according to the manufacter’s procedure on an AccuriC6 flow cytometer equipped with BD AccuriC6 software (BD, San Jose, CA). Cells were placed in suspension and 1X Dye loading solution was added for 20 min at 37 °C. Calcium concentration was then recorded for 5 min on the AccuriC6 flow cytometer. CXCR3 agonist stimulation was performed after 2 min of recording and intracellular calcium increase was visualized. Calcium concentration after stimulation was normalized by calcium concentration before stimulation. Cells without 1X dye loading solution were used as a negative control in order to quantify the background level.

### Statistical Analysis

Statistical analysis was performed using the GraphPad Prism 5 software. Multiple comparisons were performed with ANOVA analysis of variance, followed by Tukey post hoc tests.

## Electronic supplementary material

Supplementary Data File
