# Supplementary material for: Ligand activation induces different conformational changes in CXCR3 receptor isoforms as evidenced by plasmon waveguide resonance (PWR)
Source: Sci Rep. 2017 Sep 6;7:10703. doi: 10.1038/s41598-017-11151-x (PMC5587768; doi:10.1038/s41598-017-11151-x)
Supplement: Supplementary file 1 — Supplementary Data File [file 41598_2017_11151_MOESM1_ESM.pdf]

SUPPLEMENTARY DATA FILE:

Ligand activation induces different conformational changes in CXCR3 receptor isoforms as evidenced by plasmon waveguide resonance (PWR)

K. Boyé[1 2], C. Billottet[1,2], N. Pujol[1,2], I.D. Alves[3&], and A. Bikfalvi[1,2&]

[1]INSERM U1029, Pessac, France; [2]Université de Bordeaux, Pessac, France; [3]CBMN UMR 5248 CNRS, Pessac, France

Figure Legends

Supplemental Figure 1: Expression of CXCR3 in cells and membrane fragments

(A) CXCR3-A (left panel) and CXCR3-B (middle panel) mRNA expression, performed by real time PCR in parental HEK-293 cells (HEKp), HEK-CTRL, HEK-CXCR3-A and HEK-CXCR3-B clones. Values were normalized to reference gene. (B) Immunofluorescence was performed to visualize overexpressed CXCR3-A of CXCR3-B coupled to GFP. Scale: 5 µm. (C) Silencing of the overexpressed CXCR3-A or CXCR3-B was verified by immunofluorescence after transfection of human CTRL-SiRNA or CXCR3-SiRNA. (D) Silencing of CXCR3 in HEK-CXCR3-A cells using 4 different siRNA against human CXCR3 (siCXCR3-9, -10, -11 and -12). Tubulin was used as a loading control. All the results from three independent experiments were combined to calculate mean and SEM, and values were normalized to those obtained for the control, *** P<0.001, ** P<0.002. a.u. for arbitrary unit.

Supplemental Figure 2: Schematic representation of the PWR sensor

Schema of the PWR optical part with the sensor (prism that is coated with a silver and over-coated with a silica layer) and the evanescent wave (orange) that is created when light with the right energy and incident angle (α) is incident upon the back of the sensor. The sample, in this case a lipid membrane with inserted receptors (blue cylinders) and interacting ligand (red star) is placed in top of the sensor across a hole in a teflon block. The system is mounted in a rotating table allowing the incident angle to change by steps of 1 mdeg and to scan the angle at which resonance occurs that is translated by a decrease in the reflected light intensity measured by the detector.

Supplemental Figure 3: PWR spectra in the presence of agonist and antagonist

PWR spectra obtained at equilibrium upon addition of increasing concentrations of agonist (PS372424) and antagonist (SCH546738) to CXCR3-A (A and C, respectively) and CXCR3-B (B and D, respectively). Panels A and B represent spectra obtained with p-polarisation and panels C and D represent spectra obtained with s-polarisation. Spectra from the lower to higher concentration follow the shadow from black to lighter gray.

Supplemental Figure 4: Relocalisation of CXCR3-A and CXCR3-B at the cell membrane

Membrane or perinuclear localization of CXCR3-A or CXCR3-B (λ=488nm) in Hek-293 cells expressing GFP-CXCR3-A or GFP-CXCR3-B in non-stimulated conditions (NS) and upon stimulation with the CXCR3 agonist PS372424 at 15 minutes. Nuclei were stained with DAPI (λ=405nm). All sections were observed at 630× magnification under confocal laser scanning microscope (Nikon eclipse Ti). Scale: 5 μm.

Figure S1

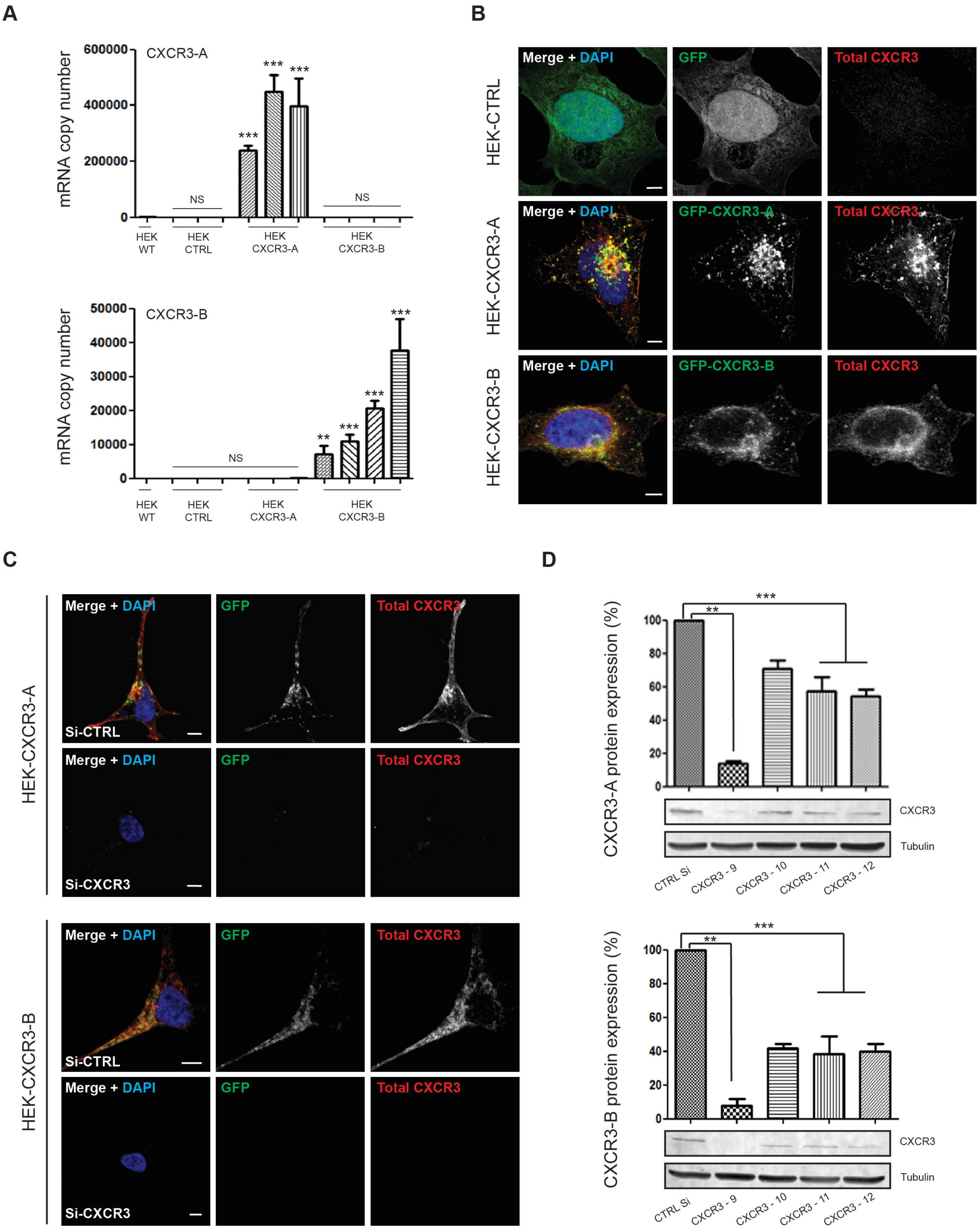

A
B
C
D
CXCR3-A
CXCR3-B
mRNA copy number
600000
400000
200000
0
50000
40000
30000
20000
10000
NS
***
**
HEK WT
HEK CTRL
HEK CXCR3-A
HEK CXCR3-B
HEK-CTRL
HEK-CXCR3-A
HEK-CXCR3-B
Merge + DAPI
GFP
Total CXCR3
GFP-CXCR3-A
GFP-CXCR3-B
Si-CTRL
Si-CXCR3
CXCR3-A protein expression (%)
CXCR3-B protein expression (%)
100
80
60
40
20
CXCR3
Tubulin
CTRL Si
CXCR3 - 9
CXCR3 - 10
CXCR3 - 11
CXCR3 - 12

Figure S2

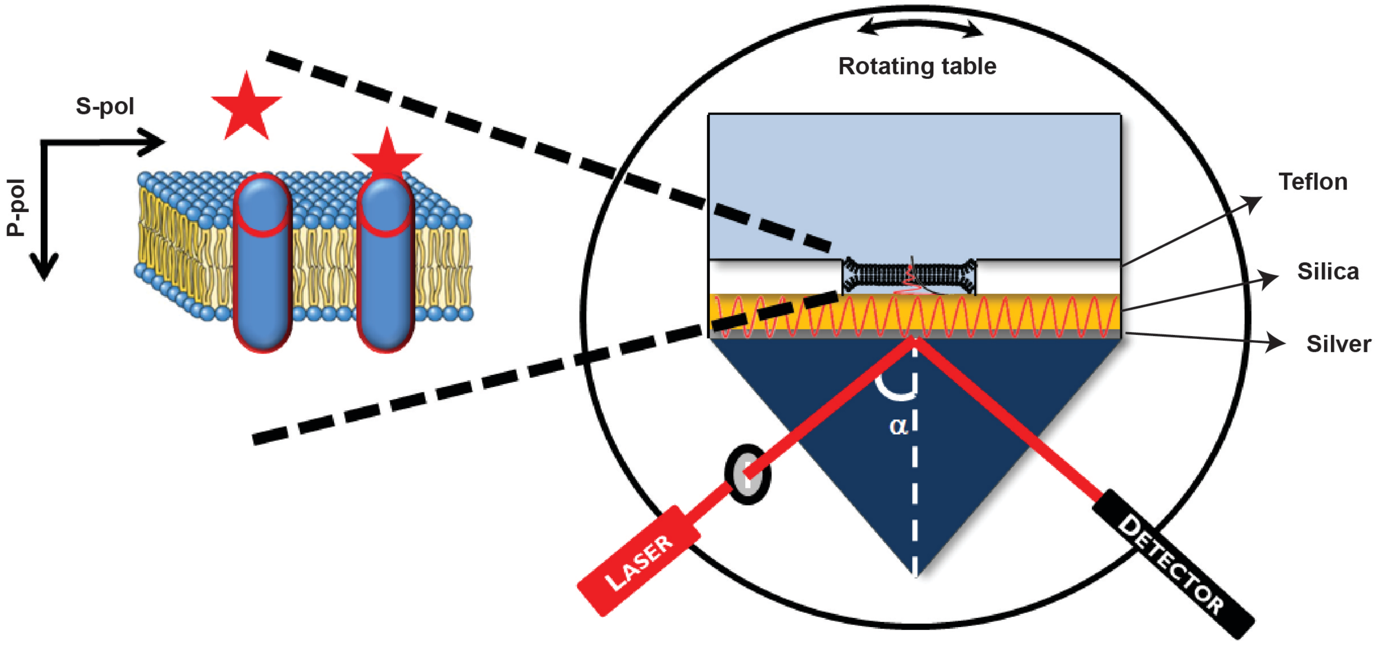

S-pol
P-pol
Rotating table
Teflon
Silica
Silver
α
LASER
DETECTOR

Figure S3

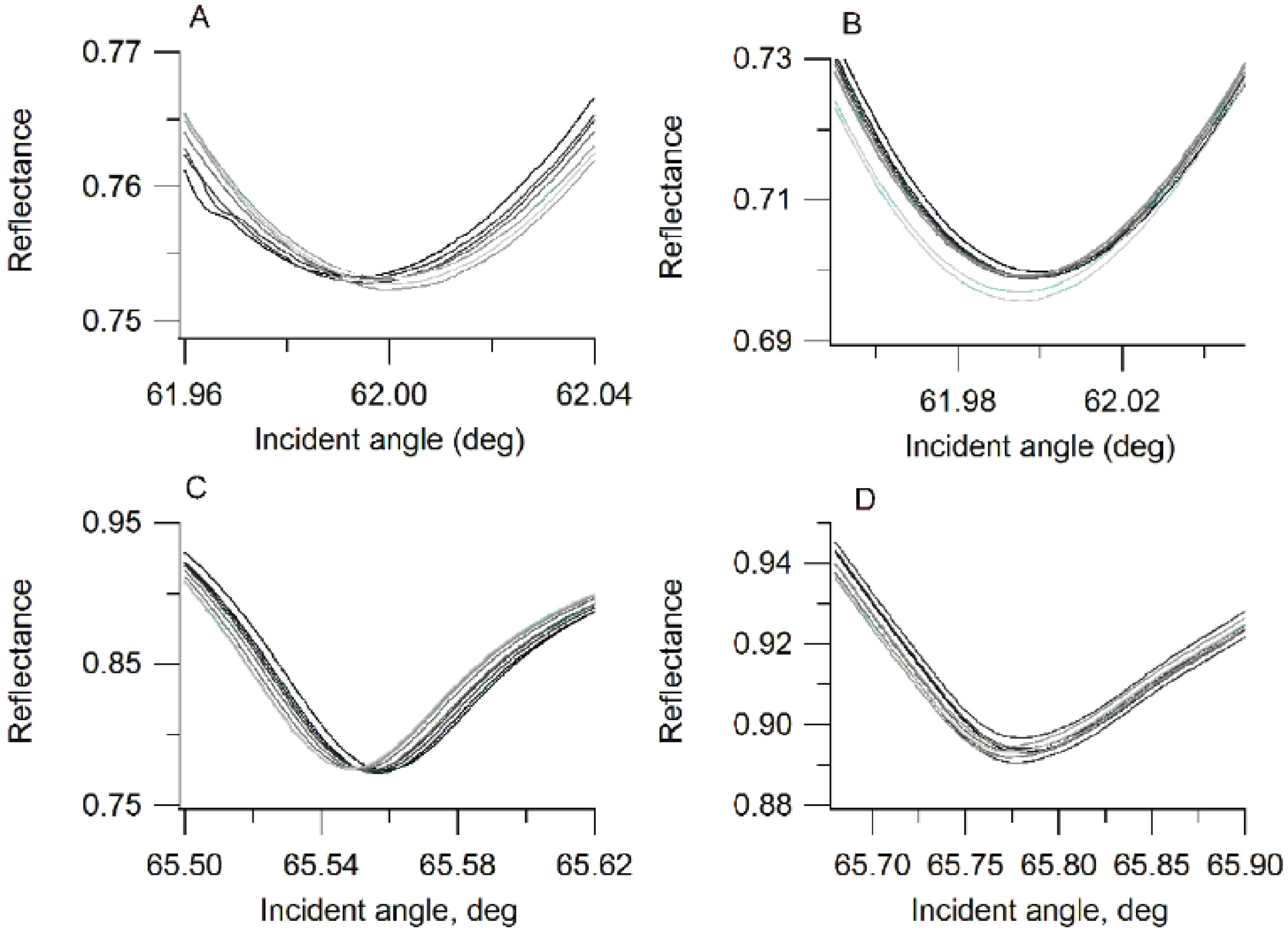

A
0.77
0.76
0.75
Reflectance
61.96
62.00
62.04
Incident angle (deg)
B
0.73
0.71
0.69
Reflectance
61.98
62.02
Incident angle (deg)
C
0.95
0.85
0.75
Reflectance
65.50
65.54
65.58
65.62
Incident angle, deg
D
0.94
0.92
0.90
0.88
Reflectance
65.70
65.75
65.80
65.85
65.90
Incident angle, deg

Figure S4

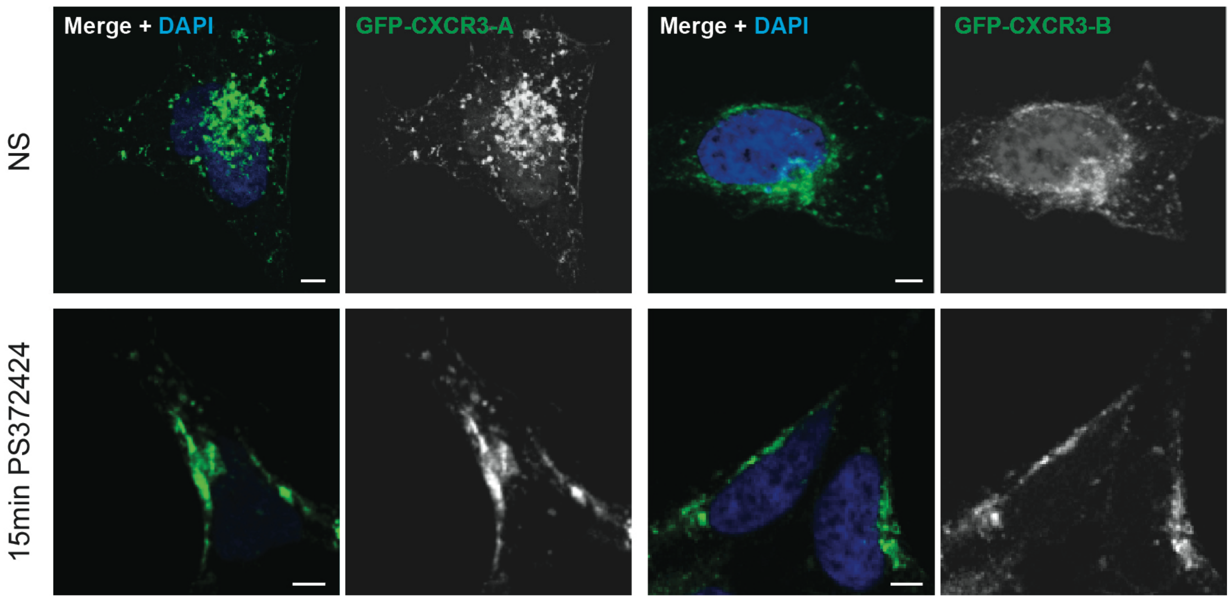

Merge + DAPI
GFP-CXCR3-A
Merge + DAPI
GFP-CXCR3-B
NS
15min PS372424
